# Binding of Small Molecules to G-quadruplex DNA in Cells Revealed by Fluorescence Lifetime Imaging Microscopy of *o*-BMVC Foci

**DOI:** 10.3390/molecules24010035

**Published:** 2018-12-21

**Authors:** Ting-Yuan Tseng, I-Te Chu, Shang-Jyun Lin, Jie Li, Ta-Chau Chang

**Affiliations:** Institute of Atomic and Molecular Sciences, Academia Sinica, Taipei 10617, Taiwan; homeotic@gmail.com (T.-Y.T.); r04223204@gmail.com (I.-T.C.); john830110@gmail.com (S.-J.L.); julielee0531@gmail.com (J.L.)

**Keywords:** G-quadruplex, G4 ligands, fluorescence lifetime imaging microscopy, *o*-BMVC foci

## Abstract

G-quadruplex (G4) structures have recently received increasing attention as a potential target for cancer research. We used time-gated fluorescence lifetime imaging microscopy (FLIM) with a G4 fluorescent probe, 3,6-bis(1-methyl-2-vinylpyridinium) carbazole diiodide (*o*-BMVC), to measure the number of *o*-BMVC foci, which may represent G4 foci, in cells as a common signature to distinguish cancer cells from normal cells. Here, the decrease in the number of *o*-BMVC foci in the pretreatment of cancer cells with TMPyP4, BRACO-19 and BMVC4 suggested that they directly bind to G4s in cells. In contrast, the increase in the number of *o*-BMVC foci in the pretreatment of cells with PDS and Hoechst 33258 (H33258) suggested that they do not inhabit the binding site of *o*-BMVC to G4s in cells. After the H33258 was removed, the gradual decrease of H33258-induced G4 foci may be due to DNA repair. The purpose of this work is to introduce o-BMVC foci as an indicator not only to verify the direct binding of potential G4 ligands to G4 structures but also to examine the possible effect of some DNA binding ligands on DNA integrity by monitoring the number of G4 foci in cells.

## 1. Introduction

G-quadruplexes (G4s) are four-stranded DNA structures that are formed by the stacking of G-quartets with Hoogsteen hydrogen bonding of four guanines under physiological conditions [1,2]. A high-throughput sequencing-based method has detected more than 700,000 G4-induced polymerase-stalling sites in the human genome [3]. However, the existence of G4s in cells has long been debated. One major concern is the chromatin integrity [4,5]. It is believed that there is very little opening for G4 formation in closed chromatin [6]. Recently, Biffi et al. [7] provided convincing evidence to illustrate the existence of G4s in cells by immunofluorescence microscopy using a G4-specific antibody BG4. In addition, more G4 foci in the pyridostatin (PDS) pretreated human U2OS cancer cells were detected using the BG4 antibody. This is because PDS can induce DNA damage at sites enriched in G4 motifs [8].

In another study, we used a G4 fluorescent probe, 3,6-bis(1-methyl-2-vinylpyridinium) carbazole diiodide (*o*-BMVC), to visualize G4s in cancer cells [9]. We took advantage of the fluorescent decay time of *o*-BMVC, which is longer upon binding to most G4s formed by G-rich sequences in telomeres and some promoter oncogenes (≥2.4 ns) than upon interaction with duplex structures such as linear duplexes and hairpin structures (~1.2 ns) [9,10]. In addition, the binding affinity of *o*-BMVC to G4 DNA is higher than to duplex DNA by nearly two orders of magnitude [9]. Very recently, we also detected elevated *o*-BMVC foci in HeLa cancer cells after PDS pretreatment by time-gated fluorescence lifetime imaging microscopy (FLIM) [10]. Of importance is that many more *o*-BMVC foci are detected in six types of cancer cells than in three types of normal cells, implying that G4s are likely a common signature of cancer cells. 

**Figure 1:**
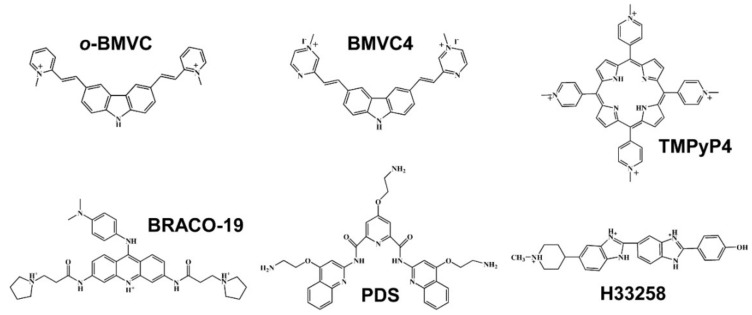
Chemical structure of six ligands.

Accumulating evidence showed that G4s are involved in a variety of biological processes [11,12,13,14,15,16,17], suggesting that G4s can act as a therapeutic target. Thus, a number of G4 ligands, such as TMPyP4 [18], BRACO-19 [19], BMVC4 [20], PDS [8], and others [21,22,23], that could induce or stabilize G4s to inhibit cancer proliferation have been considered as potential anti-cancer agents. However, it is not clear whether these G4 ligands indeed directly bind to G4s in cancer cells. For example, TMPyP4 binds with similar affinity to many other types of nucleic acid structures, although it is a good G4 stabilizer [24]. Thus, it is a preliminary requisite to verify the binding of these G4 ligands to G4s in HeLa cancer cells to validate their role as G4 binding ligands. Previously, Maiti et al. [25] reported that a modified bisbenzimide, Hoechst 33258 (H33258), can also bind to the G4 structure of human c-MYC, although H33258 is a well-known fluorescent probe for selective binding to A-T regions in the minor groove of double-stranded DNA. Here we use *o*-BMVC foci to monitor the change in the number of G4 foci in MRC-5 normal cells after adding H33258 and then terminating the use of H33258 to examine the cellular response to the binding effect of H33258. Our results suggest that the *o*-BMVC foci may act as an indicator not only for the screening of G4 binding ligands in cells but also for monitoring carcinogenic transformation. The chemical structures of the molecules studied in this work are shown in Figure 1. In addition, all DNA sequences used in this work are listed in the Supplementary Table S1.

## 2. Results

### 2.1. Study of G4 Ligands In Vitro

**Figure 2:**
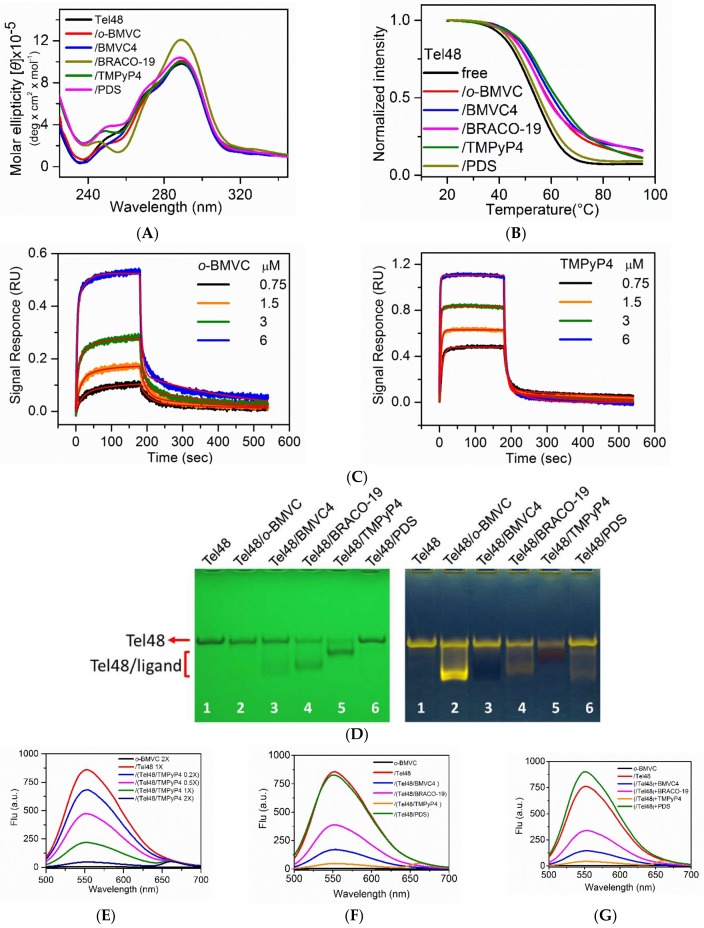

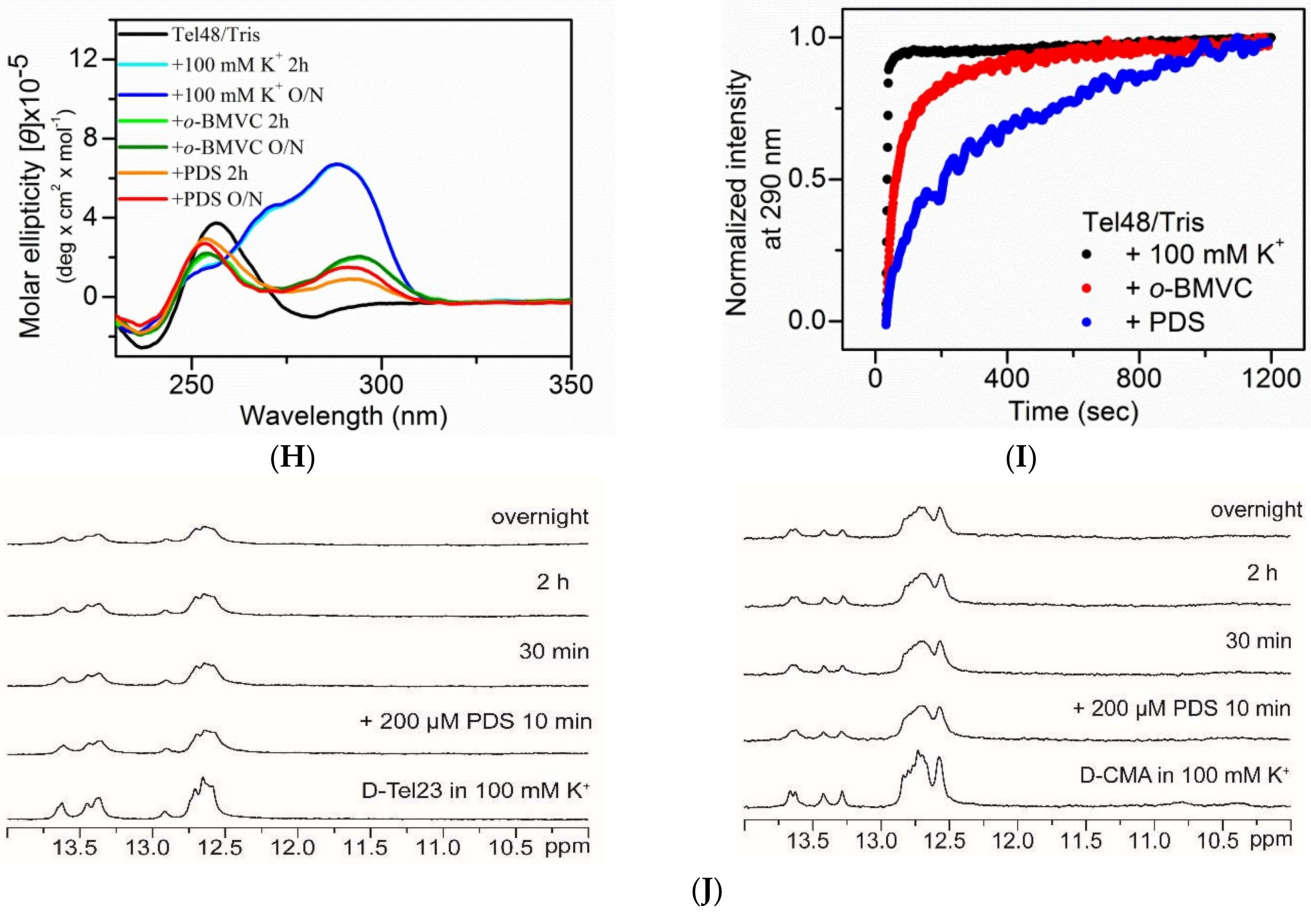
Study of G4 ligands in vitro. (**A**) The CD spectra and (**B**) CD melting curves of Tel48 in 100 mM K^+^ solution without and with *o*-BMVC, BMVC4, BRACO-19, TMPyP4 and PDS. (**C**) Kinetic binding curves for the interaction of *o*-BMVC and TMPyP4 with Tel48 fit to a two-site binding model (red lines) using the built-in software of ForteBio. The experiments were conducted in 10 mM Tris buffer with 100 mM K^+^ at 25 °C. (**D**) The UV shadowing (**left**) and post-stained by *o*-BMVC (**right**) of 40 µM Tel48 (lane 1) and its complexes with 80 µM of *o*-BMVC (lane 2), BMVC4 (lane 3), BRACO-19 (lane 4), TMPyP4 (lane 5) and PDS (lane 6). (**E**) The fluorescence spectra of 2 µM *o*-BMVC and its complexes with 1 µM Tel48, Tel48/0.2 µM TMPyP4, Tel48/0.5 µM TMPyP4, Tel48/1 µM TMPyP4 and Tel48/2 µM TMPyP4. The Tel48 sample was prepared in 100 mM K^+^ solution. (**F**) The fluorescence spectra of *o*-BMVC and its complexes with Tel48, Tel48/BMVC4, Tel48/BRACO-19, Tel48/TMPyP4 and Tel48/PDS. The concentration of Tel48 and all ligands prepared in 100 mM K^+^ solution was 1 µM and 2 µM. (**G**) The fluorescence spectra of *o*-BMVC, Tel48/*o*-BMVC, and Tel48/*o*-BMVC with the addition of BMVC4, BRACO-19, TMPyP4 and PDS, respectively. The concentration of Tel48 and all ligands prepared in 100 mM K^+^ solution was 1 µM and 2 µM. (**H**) The CD spectra of 4 µM Tel48 in Tris buffer without and with 100 mM K^+^, 8 µM *o*-BMVC and 8 µM PDS at 25 °C. (**I**) The 290 nm CD arising curves of 4 µM Tel48 with the addition of 100 mM K^+^, 8 µM *o*-BMVC and 8 µM PDS at 25 °C. (**J**) Imino proton NMR spectra of 100 µM Tel23 duplex (D-Tel23) and CMA duplex (D-CMA) in the presence of 100 mM K^+^ (bottom) and after the addition of 200 µM PDS at 10 min, 0.5 h, 2 h and overnight at 25 °C.

Circular dichroism (CD) spectra, CD melting, interferometry, gel assays, fluorescence spectra, and NMR were used to examine the interactions of Tel48 with various G4 ligands: *o*-BMVC, BMVC4, BRACO-19, TMPyP4 and pyridostatin (PDS) in vitro. We used Tel48 not only to mimic the long telomeric sequences but also to cover possible G4 topologies [26]. Similar CD spectra suggested that the binding of these molecules to Tel48 does not appreciably perturb the G4 structures (Figure 2A). The melting temperature of Tel48 G4s measured by CD melting curves showed a very slight increase of ~2 °C upon interaction with PDS, and the largest increase of ~10 °C upon interaction with TMPyP4 (Figure 2B). We further used interferometry to measure the binding parameters of these molecules to Tel48 G4s as a function of ligand concentration (Figure 2C and Figure S1). A two-site binding model [27] was applied to fit the observed binding curves resulting in two association rate constants (k_a1_ and k_a2_) and two dissociation rate constants (k_d1_ and k_d2_). The binding constant (K_b_) can be obtained from the ratio of k_a_/k_d_, where the reliable K_b_ value normally follows the criterion of 1 < K_b_ [M] < 1000 [28]. Table S2 lists the K_b_ values of these ligands to Tel48 G4s, except PDS because of insufficient signals for the reliable measurement of binding parameters. Here the results showed that TMPyP4 has the highest binding constants to Tel48 G4s. However, CD melting and interferometry studies suggested that the binding effects of PDS to G4s differ from other G4 ligands used in this work. 

Additionally, gel mobility shift provided a quick screening tool to evaluate the binding strength of these molecules to Tel48 (Figure 2D). Since the same amount of Tel48 was used in each well, one could estimate the relative intensity of the free Tel48 left in each lane (Lane 2–6) to the control lane (Lane 1) after migration. We considered the less the free Tel48 together with the more the Tel48/ligand complexes, the stronger the ligand binding. Here UV shadowing suggested that TMPyP4 (Lane 5) has much stronger G4 ligand binding strength than PDS (Lane 6) to Tel48 G4s. Moreover, the post-stained gels showed no discernible fluorescence from either the Tel48/TMPyP4 (Lane 5) or the Tel48/BMVC4 (Lane 3) complexes after post-staining with *o*-BMVC (Figure 2D), implying that TMPyP4 and BMVC4 inhabit the binding site of *o*-BMVC to G4s. Similar gel results were also observed for these G4 ligands binding to a well-defined parallel G4 structure formed by a G-rich sequence, (TAG_3_AG_3_TAG_3_AG_3_T) (CMA), originating from the 5′-end of the c-MYC promoter NHE III_1_ [29,30] (Figure S2). At present, it is not clear why Tel48/ligand bands run faster than free Tel48 bands, while CMA/ligand bands run slower than free CMA bands in the gel migration. We further measured the fluorescence spectra of the late addition of *o*-BMVC to the concentration-dependent TMPyP4 incubated with Tel48 G4s (Figure 2E). The decrease of *o*-BMVC fluorescence with the increase of TMPyP4 concentration supported our hypothesis that the binding of TMPyP4 can block the subsequent binding of *o*-BMVC to G4s.

Further gel assays and fluorescence spectra were conducted to examine whether the late addition of *o*-BMVC could replace those G4 ligands, which formerly bound to Tel48. For comparison, gel assays showed four pairs of Tel48/ligands, BMVC4, BRACO-19, TMPyP4 and PDS, without and with the late addition of *o*-BMVC for 2 h (Figure S3). We found the further decrease of free Tel48 in the presence of BMVC4, BRACO-19 and TMPyP4 together with nearly no appreciable *o*-BMVC fluorescence detected in the presence of BMVC4 and weak *o*-BMVC fluorescence detected in the presence of TMPyP4 and BRACO-19 after the late addition of *o*-BMVC. However, we did observe bright *o*-BMVC in the presence of PDS after the late addition of *o*-BMVC, suggesting that PDS did not inhabit the binding mode of *o*-BMVC to Tel48. Fluorescence spectra allowed us to quantitatively measure the fluorescence intensity of the late addition of *o*-BMVC for Tel48/ligand complexes and showed no appreciable difference for PDS, ~45% for BRACO-19,~20% for BMVC4, and ~5% for TMPyP4 to the fluorescence intensity of Tel48/*o*-BMVC (Figure 2F). In addition, fluorescence spectra were detected for the late addition of BRACO-19, BMVC4, and TMPyP4 to the Tel48/*o*-BMVC complexes (Figure 2G). It is not clear why there is no appreciable *o*-BMVC fluorescence in the presence of BMVC4 in gel assays. Of interest was the slight increase of intensity of *o*-BMVC fluorescence after the late addition of PDS, which might be due to PDS-induced G4 formation. Nevertheless, similar results of these two sets of fluorescence spectra suggested that the detection of different intensities of *o*-BMVC fluorescence was due to the equilibrium established among free Tel48, Tel48/*o*-BMVC and Tel48/ligand, implying that *o*-BMVC and these G4 ligands compete with the same binding site. This finding is important because these G4 ligands of TMPyP4, BMVC4 and BRACO-19 indeed bind to G4 structures in cells, which provides a means of screening G4 binding ligands. 

Additionally, the CD spectra showed that *o*-BMVC and PDS can induce single-stranded Tel48 to form G4s in Tris buffer solution without the presence of K^+^ (Figure 2H). However, the CD intensity was much stronger upon addition of 100 mM K^+^. The real time CD signal at 290 nm of Tel48 monitored G4 formation induced by the addition of *o*-BMVC, PDS and 100 mM K^+^ into Tris buffer solution (Figure 2I). The results suggested that the K^+^ -induced G4 formation was faster than the *o*-BMVC-induced G4 formation and much faster than the PDS-induced G4 formation. Similar results were obtained for TMPyP4 and BRACO-19 that can induce G4 formation in Tris buffer solution (Figure S4).

We further examined whether these G4 ligands can induce G4 formation from the duplex DNA. For simplicity, the NMR spectra of telomeric Tel23 duplex (D-Tel23) and CMA duplex (D-CMA) in 100 mM K^+^ solution were measured before and after the addition of two times the amount of PDS overnight (Figure 2J). The results showed the distinct signals of Watson-Crick base pairing between 12.5 and 14 ppm but no discernible G4 signals between 10.0 and 12.5 ppm of the Hoogsteen-bonded guanine bases, implying that PDS is unable to induce G4 formation from the duplex DNA in vitro. Similar NMR results were also observed after the addition of two times the amounts of TMPyP4, BRACO-19, BMVC4 or *o*-BMVC overnight (data not shown).

### 2.2. Study of G4 Ligands in Cells

After the study of these G4 ligands in vitro, it is important to examine whether these G4 ligands bind to G4 structures in cells. Very recently, we used *o*-BMVC to stain fixed cells and found a large contrast in the number of *o*-BMVC foci between cancer cells and normal cells in time-gated FLIM images for the discrimination of human cancers [10]. The details of the FLIM imaging and data analysis can be found elsewhere [10]. Briefly, typical FLIM images of fixed HeLa cancer cells incubated with *o*-BMVC were shown in Figure S5A. For simplicity, we divided the image into two temporal regions with a decay-time threshold of 2.4 ns (Figure S5B). Using the Otsu threshold method (Figure S5C) [31], the FLIM images were analyzed and separated into two channels: red (decay time ≥2.4 ns) and green (decay time <2.4 ns) (Figure S5D). Time-gated FLIM images allowed us to quantitatively measure the number of *o*-BMVC foci in fixed cells, which could act as an indicator to monitor cellular response in live cells under different conditions before the cells were fixed. 

**Figure 3:**
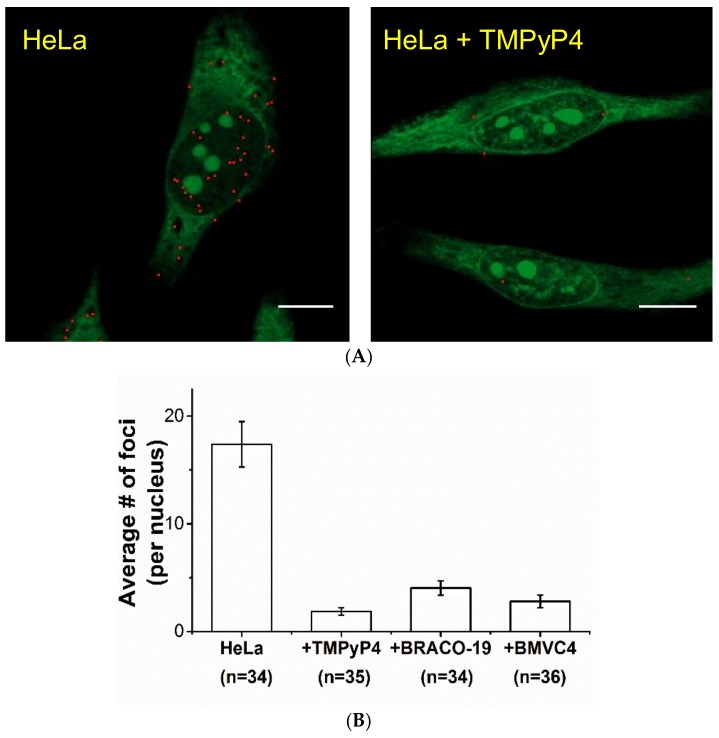
The effect of other G4 ligands on G4 foci in cells. (**A**) The analyzed binary images of *o*-BMVC foci in HeLa cancer cells (**left**) and HeLa cancer cells incubated with 10 µM of TMPyP4 overnight (**right**). These images were recorded after the cells were fixed using 70% ethanol for 10 min followed by 5 µM *o*-BMVC staining for 10 min at room temperature. Scale bar, 10 µm. The analyzed binary images were separated into two colors: red (decay time ≥2.4 ns) and green (decay time <2.4 ns). (**B**) Quantitative analyses of the average numbers of *o*-BMVC foci without and with 10 µM of TMPyP4, BRACO-19 and BMVC4 pretreatment in the fixed cells. The data obtained at least three independent experiments represent the average ± S.E.M.

Moreover, accumulating evidence suggested that the *o*-BMVC foci are mainly the G4 foci [10]. For example, the treatment of PDS ligand to HeLa cells would increase the number of G4 foci detected by G4-specific antibody BG4 [7], demonstrating that PDS traps G4 structures when they formed in cells. Consistent with their finding, time-gated FLIM images showed an appreciable increase in the number of *o*-BMVC foci for the PDS pretreated HeLa cells [10]. Since the post-stained gel results suggested that the binding of TMPyP4 can prevent the subsequent binding of *o*-BMVC to G4s, time-gated FLIM was used to measure the number of *o*-BMVC foci in HeLa cells without and with the TMPyP4 pretreatment for comparison (Figure 3A). In contrast to the results of PDS pretreatment, the images showed an appreciable decrease in the number of *o*-BMVC foci in the TMPyP4 pretreated cells. Similar results were also observed in the BRACO-19 and BMVC4 pretreated cells (data not shown). Consistent with the gel results in vitro, quantitative analyses of *o*-BMVC foci in the TMPyP4, BRACO-19 and BMVC4 pre-treated HeLa cells showed an appreciable decrease in the number of *o*-BMVC foci in the nucleus (Figure 3B), supporting our hypothesis that these G4 ligands can block the subsequent binding of *o*-BMVC to G4s. Henderson et al. [32] reported that TMPyP4 increases the number of G4 foci by immunofluorescence microscopy using a G4-specific antibody 1H6. However, they further found that the major binding site of the 1H6 antibody did not directly bind to G4s. Thus, the difference between the increase in the number of 1H6 foci and the decrease in the number of *o*-BMVC foci in the TMPyP4 pretreated HeLa cells is likely due to different binding modes of 1H6 and TMPyP4 to G4s, and the similar binding modes of *o*-BMVC and TMPyP4 to G4s.

### 2.3. Study of the Effect of Hoechst 33258 on G4 Foci in Cells

**Figure 4:**
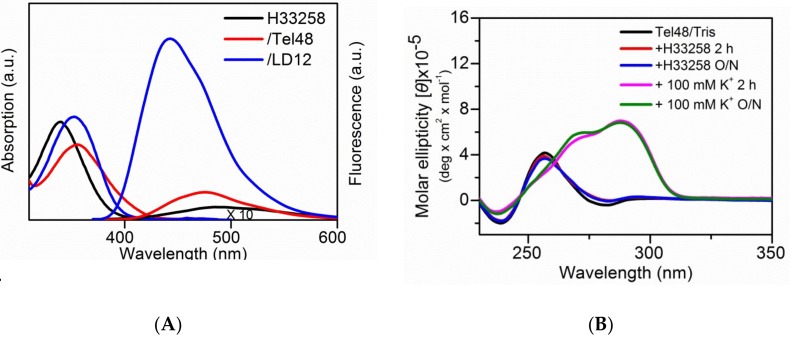

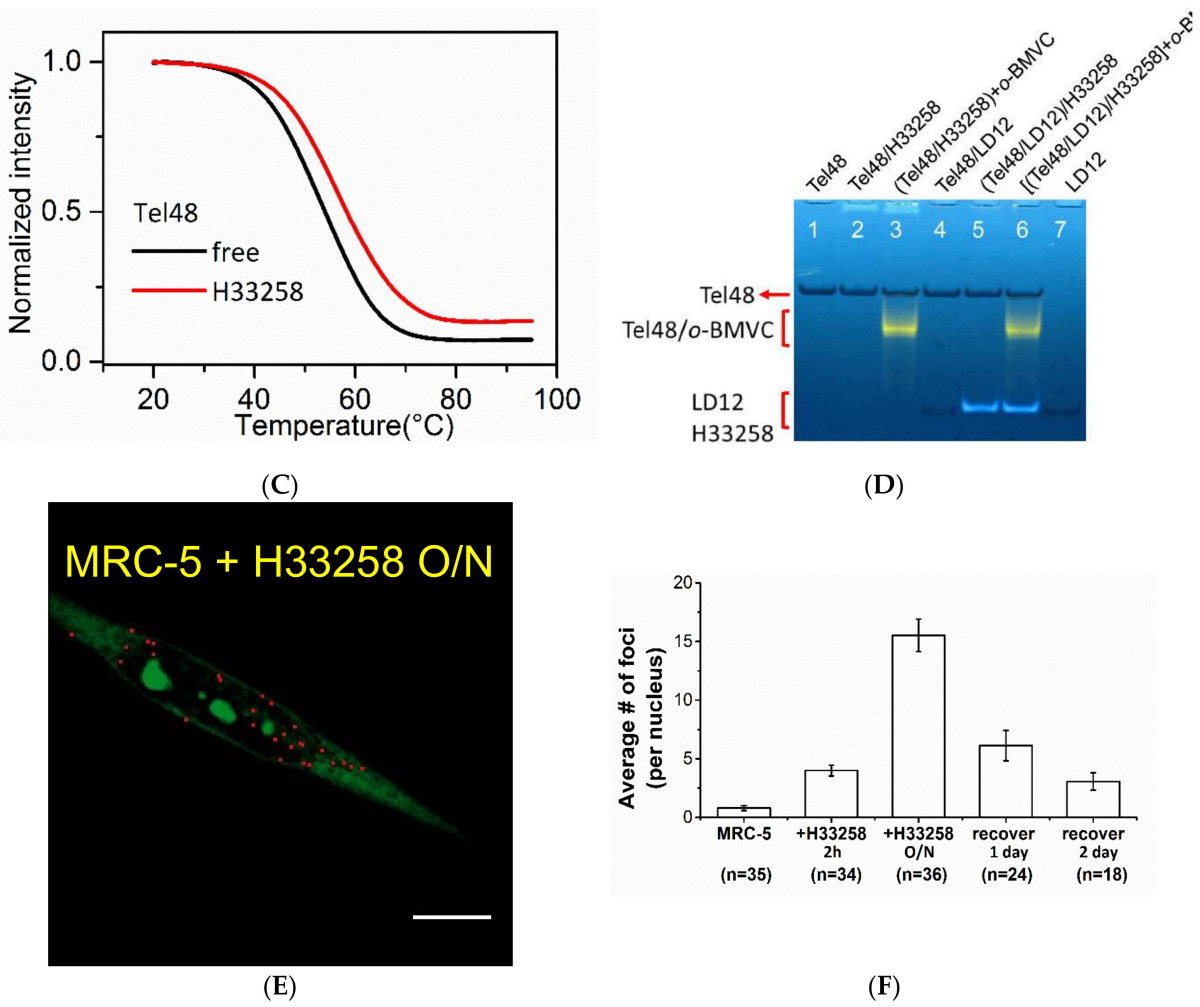
The effect of H33258 on G4 foci in cells. (**A**) The absorption and fluorescence spectra of 15 µM H33258 and its complexes with 15 µM Tel48 and 15 µM LD12. The Tel48 and LD12 sample were prepared in 100 mM K^+^ solution. (**B**) The CD spectra of 4 µM Tel48 in Tris buffer without and with 8 µM H33258 and 100 mM K^+^ at 25 °C. (**C**) The CD melting curves of Tel48 in 100 mM K^+^ solution without and with H33258. (**D**) The pre-stained gel assays of Tel48 (lane 1), Tel48/H33258 (lane 2), (Tel48/H33258) + *o*-BMVC (lane 3), Tel48 + LD12 (lane 4), (Tel48 + LD12)/H33258 (lane 5), [(Tel48 + LD12)/H33258] + *o*-BMVC (lane 6) and LD12 (lane 7). The concentration of Tel48, LD12 and two ligands prepared in 100 mM K^+^ solution were 20, 40 and 20 µM, respectively. (**E**) The analyzed binary image of *o*-BMVC foci in MRC-5 normal cells incubated with 10 µM of H33258 overnight. The image was recorded after the cells fixed using 70% ethanol for 10 min followed by 5 µM *o*-BMVC staining for 10 min at room temperature. Scale bar, 10 µm. The analyzed binary image was separated into two colors: red (decay time ≥2.4 ns) and green (decay time <2.4 ns). (**F**) Quantitative analyses of the average number of *o*-BMVC foci without H33258 and time-dependent H33258 together with the H33258 removed for 1 day and 2 days. The data obtained at least three independent experiments represent the average ± S.E.M.

Previously, Maiti et al. [25] reported that H33258 can bind to the G4 structure of human c-MYC. However, Largy et al. [33] found that H33258 clearly shows a structural preference for duplex DNA and has a lower capacity to bind G4s. Figure 4A showed the absorption and fluorescence spectra of free H33258 and its complexes with G4 DNA of Tel48 and duplex DNA of LD12 in 100 mM K^+^ solution. The fluorescence intensity of H33258 was much stronger upon interaction with LD12 duplex than with Tel48 G4s. The CD spectra showed no Tel48 G4 formation induced by H33258 in Tris buffer solution (Figure 4B). The melting temperature of Tel48 G4s showed a slight increase of ~4 °C upon interaction with H33258 (Figure 4C). However, we were not able to measure the reliable binding constants of H33258 to Tel48 because of insufficient signals in the study of interferometry. The gel assays of H33258 showed no specific effect on Tel48 G4, but clear fluorescence upon interaction with LD12 duplex (Figure 4D). In addition, the fluorescence clearly observed for the late addition of *o*-BMVC into the Tel48/H33258 mixture suggested no appreciable effect of H33258 on the binding of *o*-BMVC to Tel48 G4s.

We anticipated that the pretreatment of cells with H33258 would have less effect on the subsequent binding of *o*-BMVC to G4s. Figure 4E showed time-gated FLIM images of *o*-BMVC foci in the pretreated MRC-5 normal cells with 10 µM H33258 overnight. Quantitative analyses of the number of *o*-BMVC foci in the nucleus showed a slight increase in the pretreated MRC-5 normal cells with 10 µM H33258 for 2 h, but a marked increase in the pretreated cells with 10 µM H33258 overnight (Figure 4F). Previously, pretreatment with DNA damage by exposing MRC-5 normal cells to UV light could markedly increase the number of *o*-BMVC foci [10]. It is possible that the binding of H33258 to duplex DNA in cells may also lead to genomic instability, opening the chromatin and facilitating G4 formation from unprotected G-rich sequences. In addition, we found a gradual decrease of the induced *o*-BMVC foci after terminating UV-irradiation. The decrease of *o*-BMVC foci induced by DNA damage is probably due to the function of DNA repair [34]. Here, we also found that the number of *o*-BMVC foci induced by H33258 gradually decreases as a function of time after H33258 was removed (Figure 4F). We consider that the decrease of *o*-BMVC foci is mainly due to DNA repair [10].

## 3. Discussion

Bioimaging of fluorescent probes provides a fantastic tool to monitor target/probe interaction not only in vitro but also in cells. Our results showed the consistency of the intensity of the *o*-BMVC fluorescence in vitro with the detection of the number of *o*-BMVC foci in fixed cells due to the competition of *o*-BMVC with other G4 ligands, which allows us to monitor the binding target of G4 ligands in cells. This finding may provide a means of developing a high-throughput tool for the initial screening of G4 binding ligands based on the change of fluorescence intensity.

The increase in the number of *o*-BMVC foci in the pretreatment of cells with PDS and H33258 suggested that they may not directly bind to G4s, at least they do not inhabit the binding site of *o*-BMVC to G4s, in cells. However, they did promote G4 formation. Rodriguez et al. [8] found that PDS can induce DNA damage at sites enriched in G4 motifs. We consider that if the major binding sites of H33258 are near the region of G4 motifs, such binding may lead to genomic instability. Consequently, more opening of the total chromatin resulting from DNA damage could facilitate G4 formation from unprotected G-rich sequences. In addition, we found a gradual decrease in the number of H33258-induced *o*-BMVC foci as a function of time after H33258 was removed. Note that it is difficult to remove PDS, since PDS is quite sticky. Here, the preliminary results showed that the reducing rate of H33258-induced *o*-BMVC foci is slower than that of UV-induced *o*-BMVC foci [10], implying that different mechanisms are involved in their DNA repair. Visualizing the change of *o*-BMVC foci may apply to monitor the change from the temporary *o*-BMVC foci induced by UV-irradiation or H33258 (carcinogen) to the persistent *o*-BMVC foci in cancer cells. 

The significance of this study was to demonstrate that TMPyP4, BRACO-19 and BMVC4 directly bind to G4s in fixed cells. Given that *o*-BMVC foci represent G4 foci, the decrease in the number of G4 foci in the pretreated cells using TMPyP4, BMVC4 and BRACO-19 is due to direct binding of these G4 ligands to G4s in cells, which can prevent the subsequent binding of *o*-BMVC to G4s. This finding is very important to the development of therapeutic G4 ligands because the binding target can be identified in cells. Since the cells were fixed before the collection of FLIM data, the change of *o*-BMVC foci could act as a marker to monitor cellular response to these G4 ligands. Considering the difference between the persistent *o*-BMVC foci in cancer cells and the tentative *o*-BMVC foci induced by H33258 in normal cells, the use of *o*-BMVC foci to monitor cellular response to carcinogen may reveal the underlying mechanism of carcinogenic transformation. Such study for characterizing G4 ligands is crucial for cancer research.

## 4. Materials and Methods

### 4.1. Chemical and Sample Preparation

The synthesis of *o*-BMVC can be found elsewhere [9]. Other G4 ligands, such as PDS [8], TMPyP4 [17], BRACO-19 [18], and BMVC4 [19], have been described in the literature. All oligonucleotides purified by HPLC were purchased from Biobasic Inc. (Markham, ON, Canada). Solutions of 10 mM Tris-HCl (pH 7.5) and 100 mM KCl mixed with each oligonucleotide were heated to 95 °C for 5 min, cooled slowly at 1 °C/min to room temperature and were then stored overnight at 4 °C before use. The concentration of each oligonucleotide was determined by UV absorption nano-photometry (Implen, München, Germany).

### 4.2. Circular Dichroism (CD)

The CD spectra are the average of 5 scans on a J-815 spectropolarimeter (Jasco, Tokyo, Japan), with a 2 nm bandwidth at a 50 nm/min scan speed, as well as a 0.2 nm step resolution. The CD spectra were measured under N_2_ over the range of 230–350 nm to ascertain the G4 structures.

### 4.3. Kinetic Binding Experiment

The binding affinities of G4 ligands to Tel48 G4s were measured using label-free technology (Bio-Layer Interferometry, BLI) (ForteBio, Inc., Menlo Park, CA, USA). The experiments were performed with the Octet RED96 system equipped with super streptavidin (SSA) biosensor tips (ForteBio, Inc., Menlo Park, CA, USA). The assays were performed at 25 °C and 1000 rpm. The tips were loaded with 2 µM of biotinylated Tel48 for 5 min. The association (k_a_) and dissociation (k_d_) were established by dipping the biosensors for 10 min in various concentrations of G4 ligands dispensed in 96-microwell plates (Fisher Scientific, Turnberry Drive, Hanover Park, IL, USA) at a volume of 200 µL per well. The data were processed and analyzed using the Octet data analysis software version 7.0 (ForteBio, Inc., Menlo Park, CA, USA), and double reference subtraction was applied to eliminate background caused by buffer mismatch or insignificant non-specific binding. The results were fitted to a two-site binding model to obtain the binding constant (K_b_) from the ratio of k_a_/k_d_.

### 4.4. Nuclear Magnetic Resonance (NMR) Spectroscopy

NMR experiments were performed on Bruker AVIII 500 MHz (Bruker, Karlsruhe, Germany) equipped with a prodigy at 25 °C. The one-dimensional (1D) imino proton NMR spectra were recorded using a WATERGATE for water suppression. The DNA samples were prepared at a strand concentration of 100 µM containing 10% D_2_O in 10 mM Tris-HCl or 100 mM K^+^ salt conditions with an internal reference of 0.1 mM DSS (4,4-dimethyl-4-silapentane-1-sulfonic acid).

### 4.5. Polyacrylamide Gel Electrophoresis (PAGE)

PAGE was conducted using 20% polyacrylamide and 0.5× TBE gels. Electrophoresis was carried out at 25 mA for 3 h at 4 °C. Gels were then photographed under ultraviolet light at 254 nm using a digital camera.

### 4.6. Cell Cultures

The human normal lung fibroblast cell line MRC-5 and human cervical adenocarcinoma cell line HeLa were obtained from the American Type Culture Collection (ATCC). MRC-5 and HeLa cells were cultured in MEM medium supplemented with 10% FBS and 1% antibiotics. All cell lines were cultured in 5% CO_2_ at 37 °C. The antibiotic concentration was 100 U/mL penicillin and streptomycin.

### 4.7. Fluorescence Lifetime Imaging Microscopy (FLIM)

The setup of the FLIM system consisted of a picosecond diode laser (laser power, 5 mW) with an emission wavelength of 470 nm (LDH470; PicoQuant, Berlin, Germany) and a ~70 ps pulse width for the excitation of *o*-BMVC under a scanning microscope (IX-71 and FV-300; Olympus, Tokyo, Japan). The fluorescent signal from *o*-BMVC was collected using a 60× NA = 1.42 oil-immersion objective (PlanApoN; Olympus, Japan), passing through a 550/88 nm bandpass filter (Semrock, Rochester, NY, USA), followed by detection using a SPAD (PD-100-CTC; Micro Photon Devices, Bolzano, Italy). The fluorescence lifetime was recorded and analyzed using a time-correlated single-photon counting (TCSPC) module and software (PicoHarp 300 and SymPhoTime v5.3.2; PicoQuant, Berlin, Germany). FLIM images were constructed from pixel-by-pixel lifetime information. 

For the study of G4 ligands pretreatment, 10 µM TMPyP4, BMVC4 and BRACO-19 were used for pretreatment of HeLa cells and H33258 for pretreatment of MRC-5 cells. After washing twice, cells were fixed with 70% ethanol for 10 min and then stained with 5 µM *o*-BMVC for 10 min at room temperature. Quantitative analysis of *o*-BMVC foci by using the Otsu algorithm for the image analysis was described previously [10].

### 4.8. Quantitative Analysis of o-BMVC Foci

Since the fluorescent decay time is longer upon binding to G4s than other structures, the acquired FLIM results of *o*-BMVC in cells were presented in pseudo color and separated into two channels: white (decay time ≥2.4 ns) and red (decay time <2.4 ns) to map the G4s. Here we used HeLa cancer fixed cells as an example (Figure S5B). After excluding the non-signal pixels (intensity = 0), the gray-level histogram of the longer lifetime channel can be fitted as the mixture of Gaussians (Figure S5C). The optimal threshold was further determined by the Otsu algorithm to eliminate the weaker signals, which may be due to the loose binding of G4 DNA or the non-specific binding of small cell fragments, in the longer lifetime channel. The Otsu threshold method [31] was used to find an optimal threshold (*T*_opt_) to separate two clusters or the mixture of Gaussians, with the following formula:

$$T_{opt}=argmax\left\{\frac{P\left(T\right)\left[1-P\left(T\right)\right]{\left[m_{f}\left(T\right)-m_{b}\left(T\right)\right]}^{2}}{P\left(T\right)\sigma_{b}^{2}\left(T\right)+\left[1-P\left(T\right)\right]\sigma_{f}^{2}\left(T\right)}\right\}$$

where *P*(*T*) is the cumulative probability, *m*_b_ (*T*) is the mean of the background, *m*_f_ (*T*) is the mean of the foreground, *σ*_b_^2^(*T*) is the variance of the background and *σ*_f_^2^(*T*) is the variance of the foreground. After applying the Otsu threshold method, the weak signals can be eliminated, while the stronger signals (the red spots in Figure S5D) can be preserved. The same imaging process and analysis were applied to the FLIM images of fixed cells. By using the algorithm for the image analysis, we can lower the possible counting errors in human eye detection and unambiguously quantify the number of foci in different cell lines.

## Supplementary Materials

The following are available online, Figure S1: Kinetic binding curves for the interaction of BMVC4 and BRACO-19 with Tel48 fit to a two-site binding model (red lines) was using the built-in software of ForteBio. The experiments were conducted in 10 mM Tris buffer with 100 mM K^+^ at 25 °C., Figure S2: The UV shadowing (left) and post-stained by *o*-BMVC (right) of 40 µM CMA (lane 1) and its complexes with 80 µM of *o*-BMVC (lane 2), BMVC4 (lane 3), BRACO-19 (lane 4), TMPyP4 (lane 5) and PDS (lane 6). The CMA sample was prepared in 100 mM K^+^ solution., Figure S3: The UV shadowing (left) and post-stained by *o*-BMVC (right) of Tel48 (lane 1), Tel48/*o*-BMVC (lane 2), Tel48/BMVC4 (lane 3), (Tel48/BMVC4) + *o*-BMVC (lane 4), Tel48/BRACO-19 (lane 5), (Tel48/BRACO-19) + *o*-BMVC (lane 6), Tel48/TMPyP4 (lane 7), (Tel48/TMPyP4) + *o*-BMVC (lane 8), Tel48/PDS (lane 9) and (Tel48/PDS) + *o*-BMVC (lane 10). The concentration of Tel48 and all ligands prepared in 100 mM K^+^ solution were 40 and 80 µM, respectively., Figure S4: The CD spectra of 4 uM Tel48 in Tris buffer without and with 8 µM TMPyP4, 8 uM BRACO-19 and 100 mM K^+^ at 25 °C., Figure S5: Time-gated FLIM imaging of *o*-BMVC foci in HeLa cancer cells after fixation with 70% ethanol. FLIM images of fixed HeLa cancer cells incubated with *o*-BMVC (A). The arrow showed the long decay time. The FLIM images of fixed cells were presented in pseudo color and were separated into two components with color in white (decay time ≥2.4 ns) and in red (decay time <2.4 ns) (B). The Otsu threshold method is used to find an optimal threshold (*T*_opt_) to separate two clusters or the mixture of Gaussians in the longer lifetime channel. Typical gray-level histograms of fixed cells (C) of the longer lifetime (≥2.4 ns) channel can be fit as the mixture of Gaussians. The green lines are the Gaussian fitting curves and the red lines are the combination of fitting curves. Using the Otsu threshold method for data analysis, the weak signals can be eliminated, while the stronger signals can be preserved. The analyzed binary images of fixed cells (D) were presented in pseudo color and were separated into two components with color in red (decay time ≥2.4 ns) and in green (decay time <2.4 ns). Scale bar, 10 µm. Table S1. DNA sequences used in this work, Table S2: The binding constants of Tel48 with four ligands.
