# Supplementary material for: Binding of Small Molecules to G-quadruplex DNA in Cells Revealed by Fluorescence Lifetime Imaging Microscopy of o-BMVC Foci
Source: Molecules. 2018 Dec 21;24(1):35. doi: 10.3390/molecules24010035 (PMC6337594; doi:10.3390/molecules24010035)
Supplement: Supplementary file 1 [file molecules-24-00035-s001.pdf]

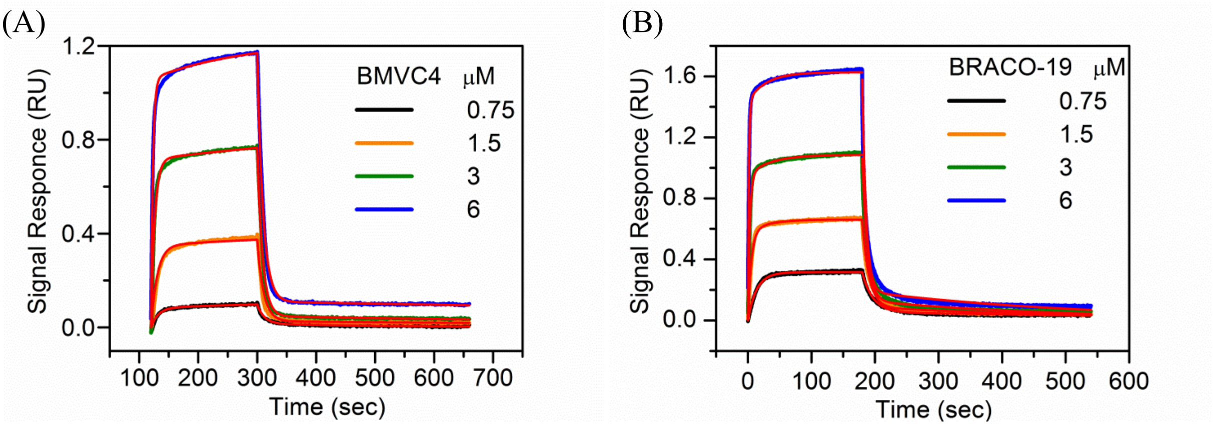

Figure S1. Kinetic binding curves for the interaction of BMVC4 and BRACO-19 with Tel48 fit to a two-site binding model (red lines) was using the built-in software of ForteBio. The experiments were conducted in 10 mM Tris buffer with 100 mM K^+ at 25 °C.

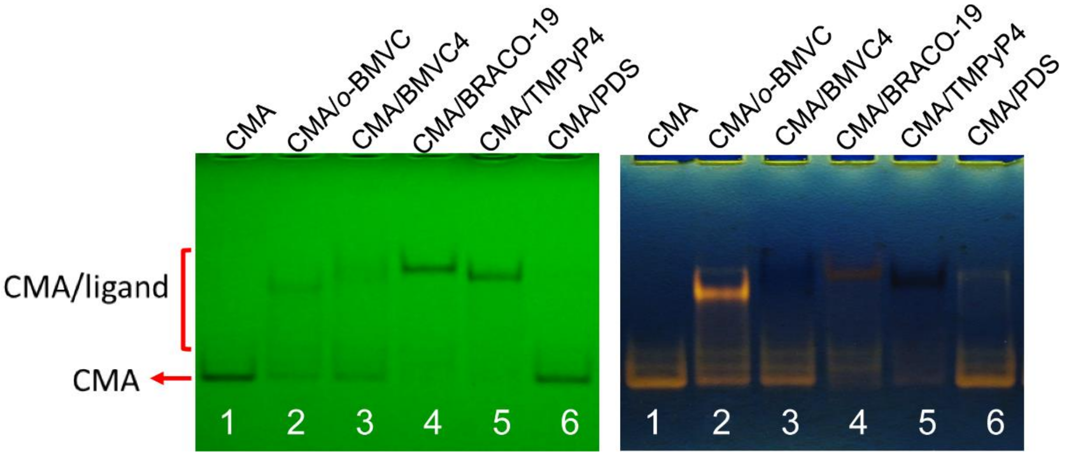

Figure S2. The UV shadowing (left) and post-stained by *o*-BMVC (right) of 40 μM CMA (lane 1) and its complexes with 80 μM of *o*-BMVC (lane 2), BMVC4 (lane 3), BRACO-19 (lane 4), TMPyP4 (lane 5) and PDS (lane 6). The CMA sample was prepared in 100 mM K^+ solution.

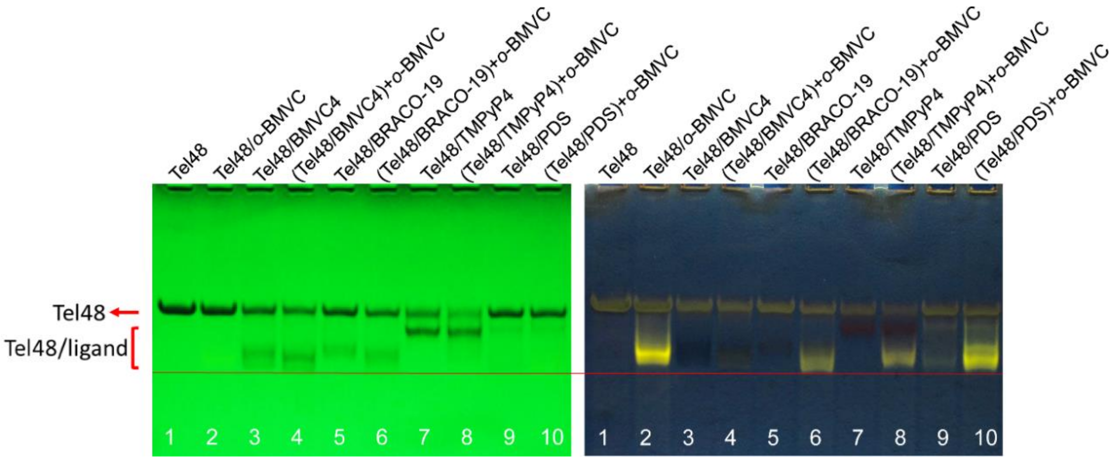

Figure S3. The UV shadowing (left) and post-stained by *o*-BMVC (right) of Tel48 (lane 1), Tel48/*o*-BMVC (lane 2), Tel48/BMVC4 (lane 3), (Tel48/BMVC4)+*o*-BMVC (lane 4), Tel48/BRACO-19 (lane 5), (Tel48/BRACO-19)+*o*-BMVC (lane 6), Tel48/TMPyP4 (lane 7), (Tel48/TMPyP4)+*o*-BMVC (lane 8), Tel48/PDS (lane 9) and (Tel48/PDS)+*o*-BMVC (lane 10). The concentration of Tel48 and all ligands prepared in 100 mM K^+ solution were 40 and 80 μM, respectively.

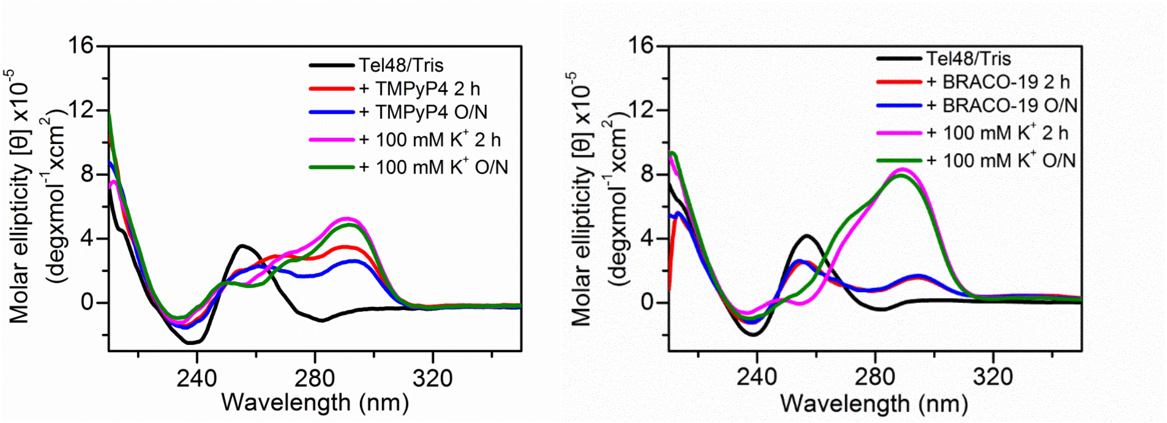

Figure S4. The CD spectra of 4 uM Tel48 in Tris buffer without and with 8 uM TMPyP4, 8 uM BRACO-19 and 100 mM K^+ at 25 °C.

(A)

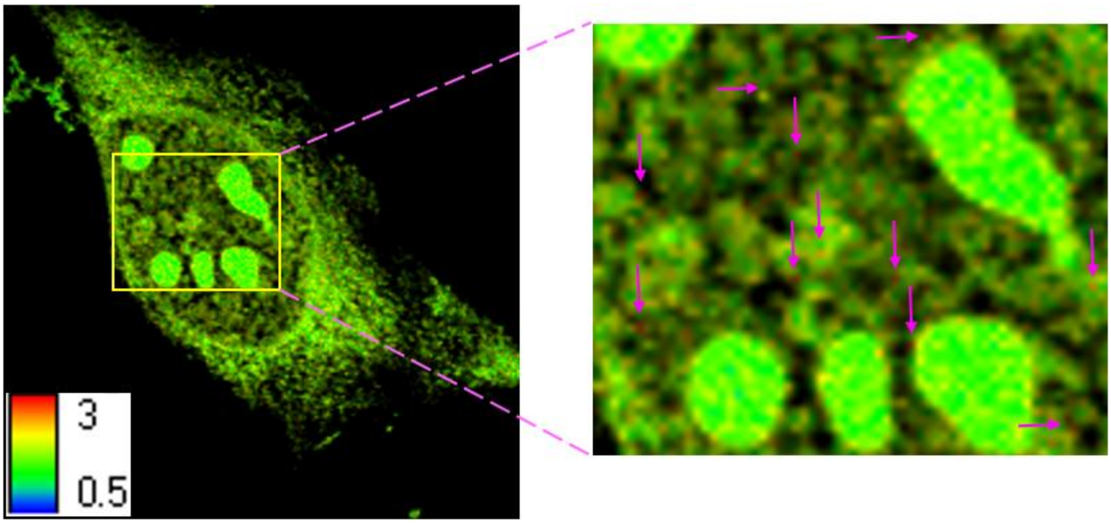

(B)

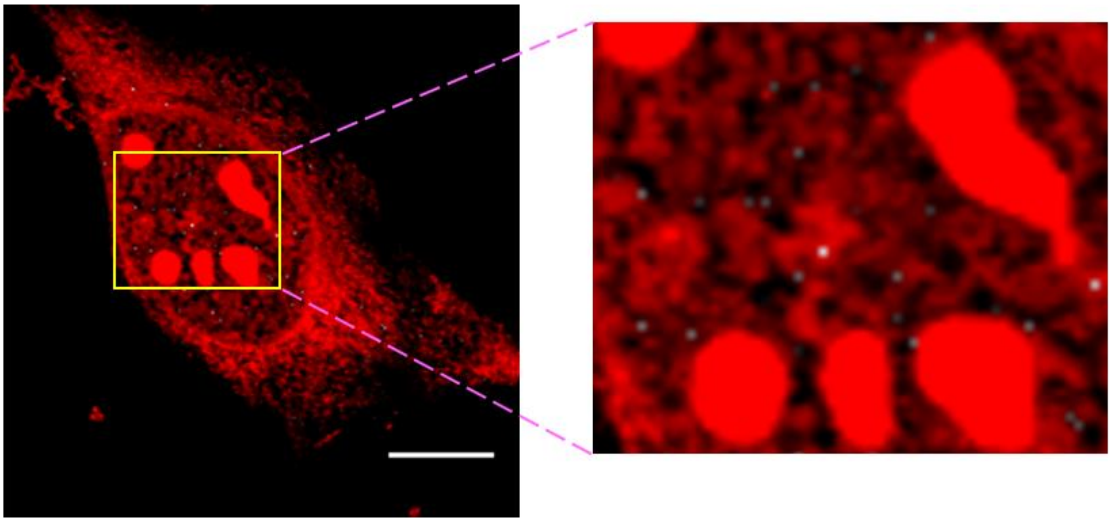

(C)

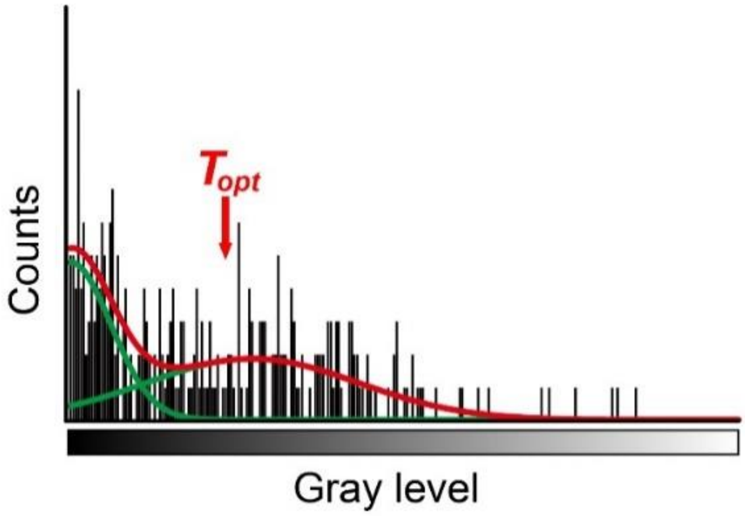

(D)

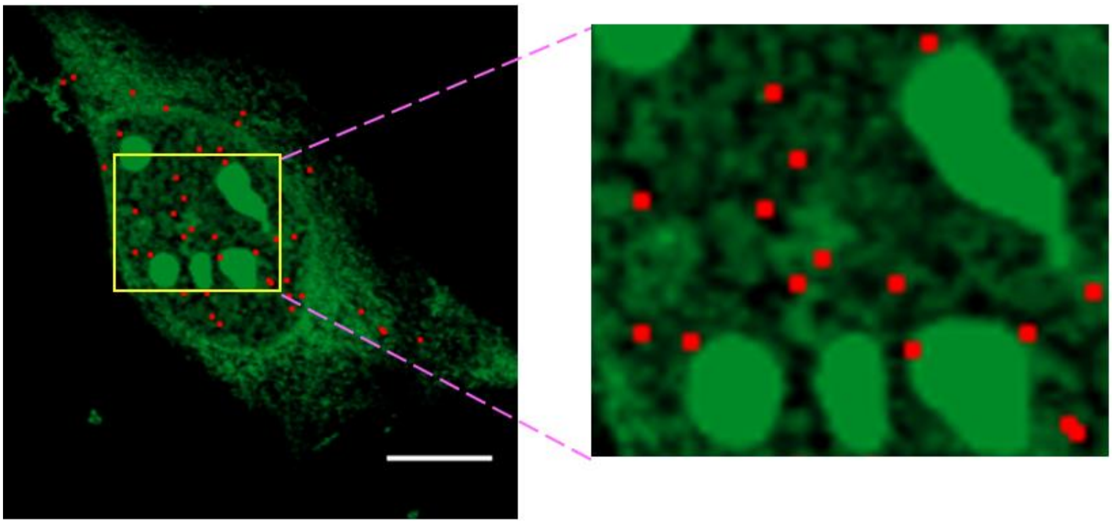

Figure S5. Time-gated FLIM imaging of *o*-BMVC foci in HeLa cancer cells after fixation with 70 % ethanol. FLIM images of fixed HeLa cancer cells incubated with *o*-BMVC (A). The arrow showed the long decay time. The FLIM images of fixed cells were presented in pseudocolor and were separated into two components with color in white (decay time ≥2.4 ns) and in red (decay time <2.4 ns) (B). The Otsu threshold method is used to find an optimal threshold (T_{opt}) to separate two clusters or the mixture of Gaussians in the longer lifetime channel. Typical gray-level histograms of fixed cells (C) of the longer lifetime (≥2.4 ns) channel can be fit as the mixture of Gaussians. The green lines are the Gaussian fitting curves and the red lines are the combination of fitting curves. Using the Otsu threshold method for data analysis, the weak signals can be eliminated, while the stronger signals can be preserved. The analyzed binary images of fixed cells (D) were presented in pseudocolor and were separated into two components with color in red (decay time ≥2.4 ns) and in green (decay time <2.4 ns). Scale bar, 10 μm.

name	Sequence
Tel48	5'-$(T_2AG_3)_8$
CMA	5'-$TAG_3AG_3TAG_3AG_3T$
LD12	5'-$GCGCA_2T_2GCGC$
D-Tel23	[5'-$TAG_3(T_2AG_3)_3$]/[$ATC_3(A_2TC_3)_3$-5']
D-CMA	[5'-$TAG_3AG_3TAG_3AG_3T$]/[$ATC_3TC_3ATC_3TC_3A$-5']

Table S1. DNA sequences used in this work

ligands	K_{b1}	k_{a1}	k_{d1}	K_{b2}	k_{a2}	k_{d2}
o-BMVC	2.38E+06	7.68E+03	3.22E-03	2.81E+05	2.50E+04	8.90E-02
TMPyP4	1.55E+07	1.32E+05	8.50E-03	4.74E+05	7.75E+04	1.63E-01
BMVC4	5.29E+05	7.88E+02	1.49E-03	1.48E+05	1.44E+04	9.78E-02
BRACO-19	9.84E+06	2.43E+04	2.74E-03	5.64E+05	6.23E+04	1.01E-01

Table S2. The binding constants of Tel48 with four ligands
